# Enhanced Antimicrobial Peptide Response Following Bacillus Calmette–Guerin Vaccination in Elderly Individuals

**DOI:** 10.3390/vaccines12091065

**Published:** 2024-09-18

**Authors:** Arul Nancy Pandiarajan, Nathella Pavan Kumar, Anuradha Rajamanickam, Perumal Kannabiran Bhavani, Bharathi Jeyadeepa, Nandhini Selvaraj, Dinesh Asokan, Srikanth Tripathy, Chandrasekharan Padmapriyadarsini, Subash Babu

**Affiliations:** 1 International Center for Excellence in Research, NIAID, Chennai 600031, India; arul.p@icerindia.org (A.N.P.); sbabu@icerindia.org (S.B.); 2 ICMR-National Institute for Research in Tuberculosis, Chennai 600031, India; 3 Laboratory of Parasitic Diseases (LPD), National Institute of Allergy and Infectious Diseases (NIAID), National Institutes of Health (NIH), Bethesda, MD 20892, USA

**Keywords:** BCG, antimicrobial peptides, tuberculosis

## Abstract

Background: Antimicrobial peptides are an important component of host defense against Mycobacterium tuberculosis. However, the ability of BCG to induce AMPs as part of its mechanism of action has not been investigated in detail. Methods: We investigated the impact of Bacillus Calmette–Guerin (BCG) vaccination on circulating plasma levels and TB-antigen stimulated plasma levels of AMPs in a healthy elderly population. We assessed the association of AMPs, including Human Beta Defensin 2 (HBD-2), Human Neutrophil Peptide 1-3 (HNP1-3), Granulysin, and Cathelicidin (LL37), in circulating plasma and TB-antigen stimulated plasma (using IGRA supernatants) at baseline (pre-vaccination) and at Month 1 and Month 6 post vaccination. Results: Post BCG vaccination, both circulating plasma levels and TB-antigen stimulated plasma levels of AMPs significantly increased at Month 1 and Month 6 compared to pre-vaccination levels in the elderly population. However, the association of AMP levels with latent TB (LTB) status did not exhibit statistical significance. Conclusion: Our findings indicate that BCG vaccination is linked to heightened circulating levels of AMPs in the elderly population, which are also TB-antigen-specific. This suggests a potential mechanism underlying the immune effects of BCG in enhancing host defense against TB.

## 1. Introduction

The Bacillus Calmette–Guérin (BCG) vaccine, made from Mycobacterium bovis, is one of the oldest and most well-known live attenuated vaccines for tuberculosis (TB), having been introduced in 1921 [1,2]. Its inclusion in the Expanded Program on Immunization (EPI) Schedule by the World Health Organization (WHO) in 1974 solidified its status as a universal vaccine, administered to over 130 million children worldwide [3], with incredible safety records [4,5]. Despite its extensive usage, TB persists as a global health challenge, with over 10 million new cases annually, leading to more than 1.3 million deaths each year [6,7,8]. Significantly, research conducted during the COVID-19 pandemic suggested a potential connection between BCG vaccination and a lower incidence of COVID-19 infection in countries with widespread BCG coverage [9,10]. Beyond its primary purpose in preventing TB, the vaccine has demonstrated efficacy in reducing mortality rates from other infections in children by approximately 50% [4,11,12]. Recognized as the sole vaccine for newborns providing TB protection up to 10 years of age, BCG’s effectiveness may extend even longer, as evidenced by studies suggesting efficacy lasting up to 20 years [3,13]. Despite its proven benefits, the vaccine’s efficacy remains suboptimal [14,15,16], prompting ongoing investigation into the immunological factors underlying its ability to enhance immunity, particularly in elderly populations.

Antimicrobial peptides (AMPs), also referred to as cationic host defense peptides (HDPs) and traditional bacterial scavengers, are emerging as a promising research focus for combating tuberculosis (TB) [17,18]. Encoded genetically, these peptides play a pivotal role in the innate immune response, boasting bactericidal activity against TB [19,20]. Due to their amphipathic nature, AMPs exhibit a range of biological activities, including disrupting microbial membranes, interfering with intracellular processes, and modulating host immune responses [18,21,22,23]. Notable among these peptides are Human Beta Defensin 2 (HBD-2), Human Neutrophil Peptide 1-3 (HNP1-3), Granulysin, and Cathelicidin (LL37), which have shown efficacy in controlling bacterial infections in in vitro studies [24,25,26,27,28]. While BCG-stimulated epithelial cells have been found to induce the mRNA expression of HBD-2 [29], the levels of AMPs in BCG-vaccinated elderly populations have not been extensively studied. The elderly population will undergo a state of dysregulated immune function due to age known as immunosenescence which is attributed to increased susceptibility to infection. *M.tb* being a thriving intracellular bacterium would tend to infect immunocompromised elderly individuals [30,31], leading to morbidity and mortality of these populations at risk. Currently, major vaccines such as the COVID-19 vaccine, influenza vaccine, pneumococcal vaccine, DTaP Vaccine (diphtheria, tetanus and pertussis), and shingles vaccine are recommended by CDC for people older than 60 years of age [32]. In a country like India, where the elderly population is often overlooked due to overpopulation and is projected to reach 179 million by 2031, proper vaccine administration is essential for their preventive care [33,34].

This study aims to investigate the influence of BCG vaccination on circulating plasma and TB-antigen-stimulated plasma levels of AMPs in healthy elderly individuals, shedding light on potential immunomodulatory effects in this demographic. In this work, we performed a detailed examination of both systemic and TB-antigen-stimulated antimicrobial peptide levels in elderly individuals before BCG vaccination and after BCG vaccination at Month 1 and Month 6. Also, within this population, the association of the AMPs between latent TB-positive and latent TB-negative individuals were analyzed.

## 2. Materials and Methods

### 2.1. Ethics Approval

Our study received approval from the ethics committee of the National Institute for Research in Tuberculosis (NIRT) with the reference number NIRT-2020010. All participants provided written informed consent before enrollment. The study is part of a clinical trial titled “Study to evaluate the effectiveness of BCG vaccine in reducing morbidity and mortality in elderly individuals in COVID-19 hotspots in India”. Additionally, the study was registered in the clinical trial registry under the identifier NCT04475302.

### 2.2. Study Population

A total of 82 participants aged between 60 and 80 years, residing in COVID-19 hotspots in Chennai, were enrolled in the study from June 2020 to October 2020 in the midst of the pandemic. Each participant received a single dose of 0.1 mL BCG vaccine (freeze-dried, manufactured by Serum Institute of India, Pune, India) intradermally over the distal insertion of the deltoid muscle on the left humerus. (The composition of the BCG vaccine was live, attenuated Bacillus Calmette–Guerin strain with every 1 mL containing between 2 × 10^6^ and 8 × 10^6^ Colony Forming Units (CFUs) with diluent: Sodium Chloride Injection I.P.) In total, 10 mL of heparinized blood and QuantiFERON(QIAGEN, Germantown, MD, USA) tubes were collected before BCG vaccination (baseline-BL) and follow-up assessments were conducted at Month 1 (M1) and Month 6 (M6) post vaccination. Heparinized whole blood was centrifuged at 2600 rpm for 10 min, and plasma was separated and aliquoted in the multiple tubes. During the screening phase for HIV and SARS-CoV-2 antibody testing, 2 mL of whole blood was collected in serum tubes. Table 1 provides a detailed demographic profile of the study population. Blood samples were collected during both the screening and enrollment phases, with screening samples obtained within two days prior to enrollment. During the screening phase, these samples were tested for SARS-CoV-2 IgG antibodies and HIV. In the enrollment phase, blood samples were collected at several time points: baseline (the day before BCG vaccination), 1 month (M1), and 6 months (M6) after vaccination. At the time of enrolment, all individuals were screened for latent TB infection (LTBI) by using QuantiFERON TB Plus; among the participants, n = 45 were LTBI-positive and n = 37 were LTBI-negative. Key exclusion criteria were elderly individuals who tested positive for SARS-CoV-2 through either antibody (serology) or PCR tests; individuals with known HIV, malignancy, or those undergoing transplantation or dialysis; those who were recently diagnosed with tuberculosis (TB) by smear or culture positivity within the previous 2 to 6 months or were receiving anti-TB treatment at the time or anti-psychiatric medications within the previous 6 months; and individuals with any contraindications to the BCG vaccine, such as allergies or hypersensitivity. The study population at baseline and M1 has been reported previously [35,36]. The most common adverse events following BCG vaccination were erythema or redness at the injection site, followed by soft swelling. Both of these effects resolved within a few days.

### 2.3. Enzyme-Linked Immunosorbent Assay (ELISA)

Circulating plasma levels and TB-antigen-specific (IGRA Supernatants) levels of antimicrobial peptides (AMPs) were measured using an Enzyme-Linked Immunosorbent Assay (ELISA). The AMPs assessed included Human Beta Defensin 2 (HBD-2), Human Neutrophil Peptide 1-3 (HNP1-3), Granulysin, and Cathelicidin (LL37). ELISA kits from Mybiosource (San Diego, CA, USA) and Hycult biotech (Wayne, PA, USA) were used for HBD2, HNP1-3, and LL37, while Granulysin was assessed using the Duoset Development System (R&D Systems). The lowest detection limits for HBD2, HNP1-3, Granulysin, and LL37 were 15.6 pg/mL, 1.56 ng/mL, 15.625 pg/mL, and 0.137 ng/mL, respectively.

### 2.4. QuantiFERON-TB Plus Plasma (Supernatant) ELISA

For analyzing the TB-antigen-specific immune response, QuantiFERON-TB Gold Plus plasma supernatants (QIAGEN, Germantown, MD, USA) were used. The QFT tubes comprised four components: NIL (for assessing immune status without stimulation), TB1 (TB1 antigen tube, which contains the ESAT-6 and CFP-10 peptide antigens to primarily detect the CD4 T-cell response), TB2 (antigen tube, which contains additional shorter peptides from ESAT-6 and CFP-10 to detect both the CD4 and CD8 T-cell responses), and mitogen (serving as a positive control). To obtain the supernatant, 1 mL of patient whole blood was incubated in these tubes according to the manufacturer’s instructions (QuantiFERON Plus Kit; Qiagen, Valencia, CA, USA). Unstimulated or TB-antigen- or mitogen-stimulated whole blood plasma (supernatants) were then used to analyze AMP levels.

### 2.5. Statistical Analysis

The concentration of antimicrobial peptides before and after (M1 and M6) BCG vaccination in the elderly population was determined using the Wilcoxon signed-rank test. The Mann–Whitney test was performed to assess statistical significance based on latent tuberculosis (LTB) status. Baseline, Month 1, and Month 6 data were represented in a single plot using one-sample *t*-tests and Wilcoxon tests, with corresponding *p*-values calculated manually. GraphPad Prism version 9.0 was utilized for data analysis.

## 3. Results

### 3.1. Elevated AMP Levels in Circulating Plasma Post BCG Vaccination

**Figure 1:**
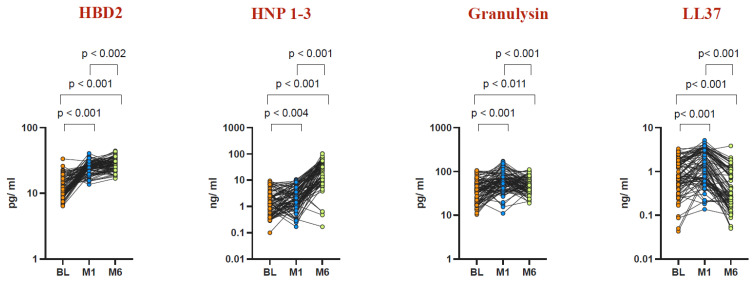
Elevated AMP levels in circulating plasma post BCG vaccination. Wilcoxon rank analysis was conducted to determine the significance level (*p*-value). Plasma levels of AMPs were measured in an elderly population (n = 82). Data representation: an orange dot represents baseline, a blue dot represents Month 1, and a light green dot represents Month 6.

To assess circulating plasma AMP levels before and after BCG vaccination, we measured the systemic levels of HBD2, HNP1-3, granulysin, and LL37 (Cathelicidin) in the elderly population, as depicted in Figure 1. Following BCG vaccination, elevated levels of HBD2, HNP1-3, granulysin, and LL37 were observed at Month 1 and Month 6 compared to baseline levels before vaccination. Specifically, at Month 1 post vaccination, the geometric mean (GM) levels of HBD2 were 24.86 pg/mL (compared to 12.34 pg/mL at baseline), those of HNP1-3 were 2.134 ng/mL (compared to 1.378 ng/mL at baseline), those of granulysin were 59.4 pg/mL (compared to 37.03 pg/mL at baseline), and those of LL37 were 1.283 ng/mL (compared to 0.786 ng/mL at baseline). Similarly, at Month 6 post vaccination, the GM levels of HBD2 were 27.06 pg/mL, those of HNP1-3 were 19.11 ng/mL, those of granulysin were 47.45 pg/mL, and those of LL37 were 0.371 ng/mL. Notably, while HBD2 and HNP1-3 exhibited a further increase at Month 6 compared to Month 1, the levels of granulysin and LL37 declined at Month 6 relative to Month 1 post vaccination.

### 3.2. No Difference in AMP Levels between LTB-Positive and -Negative Individuals (Controls)

**Figure 2:**
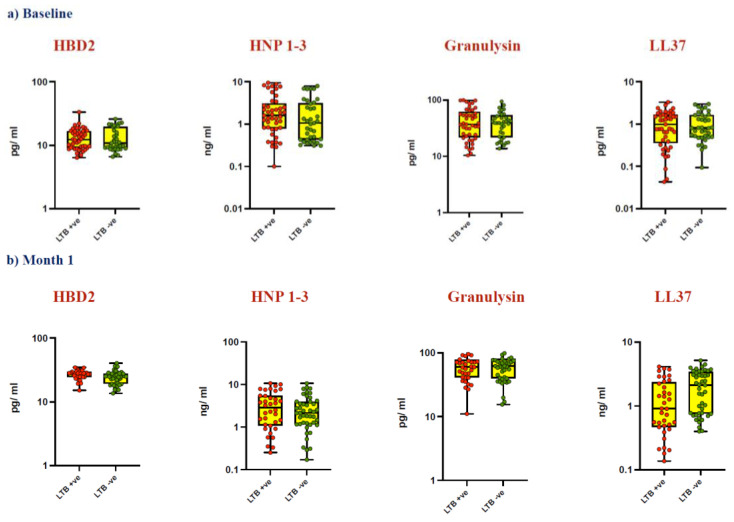

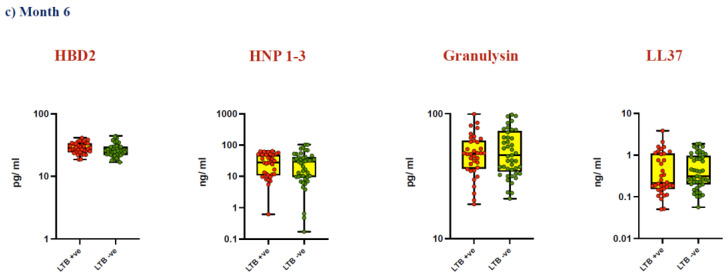
No change in AMP levels between LTB-positive and LTB-negative individuals (controls). Plasma levels of AMPs were measured in an elderly population with LTB-positive (n = 45) and LTB-negative individuals (controls) (n = 37). The Mann–Whitney test was performed to determine the significance between the two groups. Red dots represent LTB-positive, and dark green dots represent LTB-negative individuals. AMPs levels are shown at (**a**) baseline (before BCG vaccination), (**b**) Month 1 after vaccination, and (**c**) Month 6 after vaccination.

During enrolment, all participants were screened for LTBI using QuantiFERON Plus. Among them, 45 individuals tested positive for LTBI, while 37 tested negative. The levels of HBD2, HNP1-3, granulysin, and LL37 were analyzed based on IGRA status to assess any differences between LTB-positive and -negative individuals (controls) (Figure 2). Our analysis revealed no significant differences in AMP levels at baseline (Figure 2a), Month 1 (Figure 2b), and Month 6 (Figure 2c) between the two groups.

### 3.3. Elevated AMP Levels in Unstimulated Whole Blood Post BCG Vaccination

**Figure 3:**
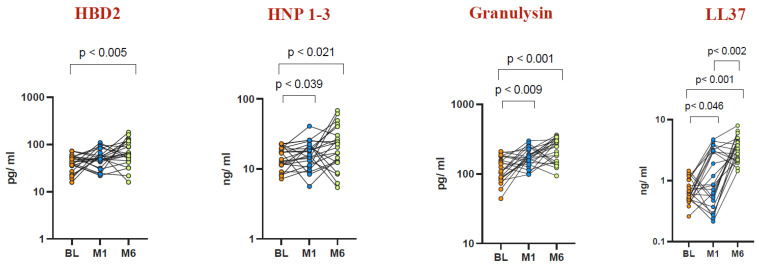
Elevated AMP levels in unstimulated whole blood at Month 1 and Month 6 post BCG vaccination compared to pre-vaccination levels. Unstimulated plasma levels of AMPs were measured in a healthy elderly population (n = 22) before vaccination (BL) and at Month 1 and Month 6 after vaccination. Wilcoxon rank analysis was performed to determine the significance level (*p*-value).

To evaluate unstimulated AMP levels before and after BCG vaccination at Month 1 and Month 6, we measured the levels of HBD2, HNP1-3, granulysin, and LL37 (Figure 3). Elevated levels of HBD2 were observed at Month 6 post vaccination (GM of 63.85 pg/mL vs. baseline GM of 40.18 pg/mL, *p* < 0.005), while HNP1-3 exhibited increased levels at both Month 1 (GM of 15.86 ng/mL vs. baseline GM of 14.36 ng/mL, *p* < 0.039) and Month 6 (GM of 16.18 ng/mL vs. baseline GM of 14.36 ng/mL, *p* < 0.021) compared to baseline. Furthermore, granulysin levels were elevated at Month 1 (GM of 172.6 pg/mL vs. baseline GM of 116.9 pg/mL) and Month 6 (GM of 184.4 pg/mL vs. baseline GM of 116.9 pg/mL, *p* < 0.001) post vaccination. LL37 levels also showed an increase at Month 1 (GM of 1.082 ng/mL vs. baseline GM of 0.61 ng/mL, *p* < 0.046) and Month 6 compared to baseline. Notably, LL37 levels at Month 6 post vaccination (GM of 3.292 ng/mL) were higher than both Month 1 (GM of 1.082 ng/mL) and baseline (GM of 0.61 ng/mL) levels.

### 3.4. Increased AMP Net Levels in TB-Antigen 1- and TB-Antigen 2-Stimulated Whole Blood Post BCG Vaccination

**Figure 4:**
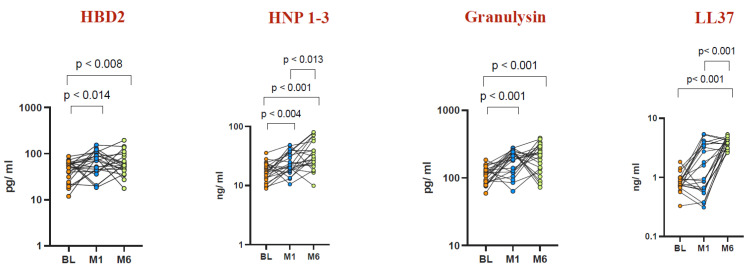
Elevated AMP levels in TB-Antigen 1-stimulated whole blood at Month 1 and Month 6 post BCG vaccination compared to pre-vaccination levels. TB-Antigen 1-stimulated plasma levels of AMPs were measured in a healthy elderly population (n = 22) before vaccination (BL) and at Month 1 and Month 6 after vaccination. Wilcoxon rank analysis was conducted to determine the significance level (*p*-value).

**Figure 5:**
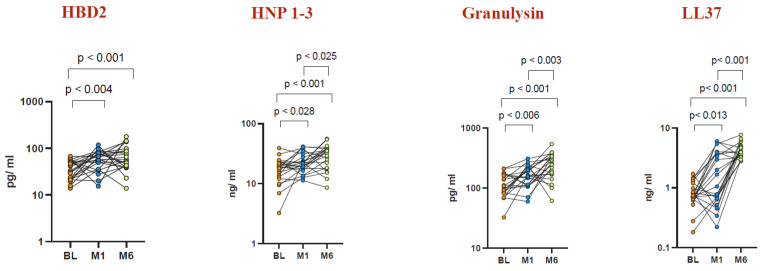
Elevated AMP levels in TB-Antigen 2-stimulated whole blood at Month 1 and Month 6 post BCG vaccination compared to pre-vaccination levels. TB-Antigen 2-stimulated plasma levels of AMPs were measured in a healthy elderly population (n = 22) before vaccination (BL) and at Month 1 and Month 6 after vaccination. Wilcoxon rank analysis was performed to determine the significance level (*p*-value).

To assess the impact of BCG vaccination on TB-antigen-stimulated AMP net levels, we measured the concentrations of HBD2, HNP1-3, granulysin, and LL37 (Figure 4 for TB-Antigen 1 and Figure 5 for TB-Antigen 2). Elevated levels of AMPs were observed at both Month 1 and Month 6 post vaccination compared to baseline levels. For TB-Antigen 1 stimulation, HBD2 levels were significantly elevated at Month 1 (GM of 60.67 pg/mL vs. baseline GM of 42.76 pg/mL, *p* < 0.014) and Month 6 (GM of 59.96 pg/mL vs. baseline GM of 42.76 pg/mL, *p* < 0.008). Similarly, HNP1-3 levels showed a significant increase at Month 1 (GM of 23.93 ng/mL vs. baseline GM of 16.97 ng/mL, *p* < 0.004) and Month 6 (GM of 32.96 ng/mL vs. baseline GM of 16.97 ng/mL, *p* < 0.001). Granulysin levels also exhibited a significant elevation at Month 1 (GM of 171.4 pg/mL vs. baseline GM of 108.4 pg/mL, *p* < 0.001) and Month 6 (GM of 178.5 pg/mL vs. baseline GM of 108.4 pg/mL, *p* < 0.001). LL37 levels were notably increased at Month 6 post vaccination (GM of 3.79 ng/mL vs. baseline GM of 0.83 ng/mL, *p* < 0.001).

Similarly, for TB-Antigen 2 stimulation, HBD2 net levels showed significant elevation at Month 1 (GM of 54.28 pg/mL vs. baseline GM of 34.14 pg/mL, *p* < 0.004) and Month 6 (GM of 60.92 pg/mL vs. baseline GM of 34.14 pg/mL, *p* < 0.001). HNP1-3 levels were also significantly increased at Month 1 (GM of 21.83 ng/mL vs. baseline GM of 16.3 ng/mL, *p* < 0.028) and Month 6 (GM of 27.69 ng/mL vs. baseline GM of 16.3 ng/mL, *p* < 0.001). Granulysin levels exhibited a significant elevation at Month 1 (GM of 157.3 pg/mL vs. baseline GM of 109.7 pg/mL, *p* < 0.006) and Month 6 (GM of 187.2 pg/mL vs. baseline GM of 109.7 pg/mL, *p* < 0.001). LL37 levels also showed a significant increase at both Month 1 (GM of 1.309 ng/mL vs. baseline GM of 0.754 ng/mL, *p* < 0.013) and Month 6 (GM of 4.194 ng/mL vs. baseline GM of 0.754 ng/mL, *p* < 0.001).

### 3.5. No Change in AMP Net Levels in Mitogen-Stimulated Whole Blood Post BCG Vaccination

**Figure 6:**
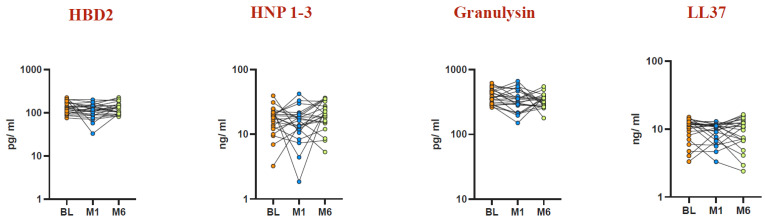
No change in AMP levels in mitogen-stimulated whole blood before BCG vaccination and at Month 1 and Month 6 after vaccination. Mitogen-stimulated plasma levels of AMPs were measured in a healthy elderly population (n = 22) before vaccination (BL) and at Month 1 and Month 6 after vaccination. Wilcoxon rank analysis was conducted to determine the significance level (*p*-value).

In contrast, there were no significant differences observed in AMP levels upon mitogen stimulation before BCG vaccination and at Month 1 and Month 6 post vaccination (Figure 6).

## 4. Discussion

Antimicrobial peptides (AMPs) interact with host cells by either penetrating them or modulating the host immune response. Various published studies have underscored the significance of AMPs in the host’s defense against tuberculosis (TB) [37]. Additionally, several studies have demonstrated the effectiveness of AMPs in promoting autophagy for bacterial elimination [37]. These small biomolecules, consisting of 20 to 60 amino acids, are renowned for their role in the innate immune response and could be administered alongside other anti-TB treatments. They exhibit high anti-mycobacterial activity with low immunogenicity, making them promising therapeutic agents for TB [17,38]. Despite their potential, few studies have investigated the impact of treatment on the systemic levels of AMPs in active TB disease [23]. AMPs primarily eliminate pathogens by disrupting the physical integrity of microbial membranes or by translocating across membranes into bacterial cytoplasm to target intracellular components [18]. In vitro studies have demonstrated that peptides like LL37 inhibit the growth of mycobacteria such as *M. bovis* BCG, *M. smegmatis*, and H37Rv by enhancing the co-localization of *M.tb* with lysosomes within phagosomes. However, several studies suggest that AMPs are weakly induced in *M.tb*-infected macrophages [25,39,40].

Elderly individuals are particularly vulnerable to infectious diseases and other health conditions due to inflammaging and immune senescence [41,42,43]. BCG vaccination continues to be regarded as safe, with very rare adverse effects observed, particularly in the Brazilian population, where the second dose has shown minimal adverse effects compared to the first dose [44]. Previous studies have reported vaccine efficacy of 9% up to five years and 12% up to nine years of follow-up in children aged between 7 and 14 [45,46].

Our previous investigations within the same cohort aimed at assessing the impact of BCG vaccination on COVID-19 susceptibility revealed that vaccinated individuals exhibited decreased plasma levels of cytokines, chemokines, acute phase proteins (APPs), matrix metalloproteinases (MMPs), and growth factors [35]. Furthermore, in individuals with active tuberculosis and comorbidities such as diabetes mellitus (DM), elevated levels of HNP1-3, HBD2, and LL37, along with decreased levels of granulysin, were observed compared to individuals without DM. Notably, anti-TB treatment reversed the AMP levels in both PTB-DM and PTB groups [47]. Additionally, our subsequent investigations indicated that BCG vaccination not only increased the frequencies of plasmacytoid and myeloid dendritic cells but also altered the levels of type I and type III interferons, suggesting its potential to induce a non-specific innate immune response [36]. Moreover, BCG vaccination was found to enhance B cell subsets, indicating its role in augmenting heterologous immunity [48,49].

Our results indicate that BCG vaccination boosts the levels of antimicrobial peptides (AMPs)—including HNP1-3, HBD2, LL37, and granulysin—in both circulating plasma and plasma samples stimulated with TB antigens in healthy elderly individuals (Figure 1, Figure 3, Figure 4 and Figure 5). This implies that BCG vaccination not only boosts general innate immune responses but also triggers the production of antimicrobial peptides (AMPs) specific to TB antigens (Figure 4 and Figure 5). Furthermore, BCG has been associated with an increase in memory T cell subsets and a strengthening of γC cytokine responses, suggesting its capacity to promote broad adaptive immune responses [50]. Interestingly, there was no statistically significant difference observed in AMP levels upon mitogen stimulation before BCG vaccination and at Months 1 and 6 post vaccination. This suggests that the elevated AMP response induced by BCG vaccination is primarily driven by TB antigens rather than by mitogen stimulation.

Diminished systemic and *M.tb* antigen-specific levels of HBD2, HNP1-3, LL37, and granulysin have been observed in latent tuberculosis (LTB) individuals with low body mass index (BMI), indicating a heightened risk of progression to active disease [51]. However, in contrast to these findings, our data did not show any statistically significant differences in AMP levels between LTB-positive and LTB-negative populations at baseline before BCG vaccination, nor at Month 1 and Month 6 post vaccination (Figure 2). This lack of significance may be attributed to confounding factors such as low BMI, which is strongly associated with immune system dysregulation [52,53].

A notable strength of our study is the follow-up of elderly participants for up to 6 months after BCG vaccination. However, a major limitation is the absence of a placebo control group or an unvaccinated group, which could have provided valuable comparative data. In summary, our study clearly demonstrates that circulating plasma levels and TB-antigen-stimulated plasma levels of AMPs such as HBD2, HNP1-3, LL37, and granulysin were significantly elevated at Month 1 and Month 6 post BCG vaccination compared to pre-vaccination levels in the elderly population. Notably, the association of AMP levels with LTB status did not show any statistical significance. These findings underscore the association of BCG vaccination with enhanced circulating levels of AMPs in the elderly population, which are also TB-antigen-specific and independent of LTB status. Further exploration of these findings could aid in validating the efficacy of the vaccine by determining the break point of pulmonary tuberculosis by following up the current elderly population with a cross sectional study, and exploring novel applications for the BCG shot.

## 5. Conclusions

Our research has revealed that BCG vaccination is associated with increased levels of antimicrobial peptides in the blood of elderly individuals. Notably, these elevated AMP levels are specific to tuberculosis antigens. This observation suggests a possible mechanism by which the BCG vaccine enhances the immune system’s ability to defend itself against TB, particularly in older adults.

## Figures and Tables

**Table 1:** Demographics of the elderly population.

S. No	Parameters	Baseline (n = 82)
1	Age (years), Median [IQR]	65 (61, 70)
2	Gender (M/F), n	53/29
3	Height (cm), Median (IQR)	159 (151.0, 165.8)
4	Weight (Kg), Median (IQR)	56.55 (52.62, 62.48)
5	Pulse Rate, Median (IQR)	88.50 (82, 95)
6	Systolic Blood Pressure, Median (IQR)	130 (120, 146)
7	Diastolic Blood Pressure, Median (IQR)	80 (70, 90)
8	SPOS%, Median (IQR)	97 (97, 98)
9	Smoking, n (%)	9 (8)
10	Alcoholism, n (%)	8 (8)
11	Diabetes Mellitus (%)	36 (43)
12	Cardiovascular Disease, n (%)	15 (18)
13	Respiratory Disease, n (%)	12 (14)
14	Musculoskeletal Disease, n (%)	1 (1)
15	Gastrointestinal Disease, n (%)	1 (1)
16	Endocrine Disease, n (%)	24 (28)
17	Dermatological Disease, n (%)	17 (20)
18	Allergies, n (%)	2 (1)
19	Neurological Disease, n (%)	1 (1)
20	LTBI Status, n (%)	Positive—45 (55)
		Negative—37 (45)

All required data were captured only during baseline; no specific data were collected during the follow-up time points.

## Data Availability

All the reported data are available within the manuscript.

## References

[1] Calmette A. (1931). Preventive Vaccination Against Tuberculosis with BCG. Proc. R. Soc. Med..

[2] Luca S., Mihaescu T. (2013). History of BCG Vaccine. Maedica.

[3] Abubakar I., Pimpin L., Ariti C., Beynon R., Mangtani P., Sterne J.A., Fine P.E., Smith P.G., Lipman M., Elliman D. (2013). Systematic review and meta-analysis of the current evidence on the duration of protection by bacillus Calmette-Guerin vaccination against tuberculosis. Health Technol. Assess..

[4] McShane H. (2011). Tuberculosis vaccines: Beyond bacille Calmette-Guerin. Philos. Trans. R. Soc. Lond. B Biol. Sci..

[5] Ottenhoff T.H., Kaufmann S.H. (2012). Vaccines against tuberculosis: Where are we and where do we need to go?. PLoS Pathog..

[6] Zwerling A., Behr M.A., Verma A., Brewer T.F., Menzies D., Pai M. (2011). The BCG World Atlas: A database of global BCG vaccination policies and practices. PLoS Med..

[7] Kuan R., Muskat K., Peters B., Lindestam Arlehamn C.S. (2020). Is mapping the BCG vaccine-induced immune responses the key to improving the efficacy against tuberculosis?. J. Intern. Med..

[8] WHO (2023). Global Tuberculosis Report 2023.

[9] Sinha S., Ajayababu A., Thukral H., Gupta S., Guha S.K., Basu A., Gupta G., Thakur P., Lingaiah R., Das B.K. (2022). Efficacy of Bacillus Calmette-Guerin (BCG) Vaccination in Reducing the Incidence and Severity of COVID-19 in High-Risk Population (BRIC): A Phase III, Multi-centre, Quadruple-Blind Randomised Control Trial. Infect. Dis. Ther..

[10] Madan M., Pahuja S., Mohan A., Pandey R.M., Madan K., Hadda V., Tiwari P., Guleria R., Mittal S. (2020). TB infection and BCG vaccination: Are we protected from COVID-19?. Public Health.

[11] Shann F. (2013). Nonspecific effects of vaccines and the reduction of mortality in children. Clin. Ther..

[12] Benn C.S., Netea M.G., Selin L.K., Aaby P. (2013). A small jab—A big effect: Nonspecific immunomodulation by vaccines. Trends Immunol..

[13] Mangtani P., Nguipdop-Djomo P., Keogh R.H., Sterne J.A.C., Abubakar I., Smith P.G., Fine P.E.M., Vynnycky E., Watson J.M., Elliman D. (2018). The duration of protection of school-aged BCG vaccination in England: A population-based case-control study. Int. J. Epidemiol..

[14] Qu M., Zhou X., Li H. (2021). BCG vaccination strategies against tuberculosis: Updates and perspectives. Hum. Vaccin. Immunother..

[15] Dockrell H.M., Butkeviciute E. (2022). Can what have we learnt about BCG vaccination in the last 20 years help us to design a better tuberculosis vaccine?. Vaccine.

[16] Lawrence A. (2024). Bacillus Calmette-Guerin (BCG) Revaccination and Protection Against Tuberculosis: A Systematic Review. Cureus.

[17] AlMatar M., Makky E.A., Yakici G., Var I., Kayar B., Koksal F. (2018). Antimicrobial peptides as an alternative to anti-tuberculosis drugs. Pharmacol. Res..

[18] Hancock R.E., Sahl H.G. (2006). Antimicrobial and host-defense peptides as new anti-infective therapeutic strategies. Nat. Biotechnol..

[19] Zasloff M. (2002). Antimicrobial peptides in health and disease. N. Engl. J. Med..

[20] Lehrer R.I., Ganz T. (2002). Defensins of vertebrate animals. Curr. Opin. Immunol..

[21] Oliveira G.S., Costa R.P., Gomes P., Gomes M.S., Silva T., Teixeira C. (2021). Antimicrobial Peptides as Potential Anti-Tubercular Leads: A Concise Review. Pharmaceuticals.

[22] Padhi A., Sengupta M., Sengupta S., Roehm K.H., Sonawane A. (2014). Antimicrobial peptides and proteins in mycobacterial therapy: Current status and future prospects. Tuberculosis.

[23] Hancock R.E., Haney E.F., Gill E.E. (2016). The immunology of host defence peptides: Beyond antimicrobial activity. Nat. Rev. Immunol..

[24] Bals R., Wilson J.M. (2003). Cathelicidins--a family of multifunctional antimicrobial peptides. Cell Mol. Life Sci..

[25] Rivas-Santiago B., Hernandez-Pando R., Carranza C., Juarez E., Contreras J.L., Aguilar-Leon D., Torres M., Sada E. (2008). Expression of cathelicidin LL-37 during Mycobacterium tuberculosis infection in human alveolar macrophages, monocytes, neutrophils, and epithelial cells. Infect. Immun..

[26] Rivas-Santiago B., Sada E., Hernandez-Pando R., Tsutsumi V. (2006). Antimicrobial peptides in the innate immunity of infectious diseases. Salud Publica Mex..

[27] Rivas-Santiago B., Serrano C.J., Enciso-Moreno J.A. (2009). Susceptibility to infectious diseases based on antimicrobial peptide production. Infect. Immun..

[28] Silva J.P., Appelberg R., Gama F.M. (2016). Antimicrobial peptides as novel anti-tuberculosis therapeutics. Biotechnol. Adv..

[29] Mendez-Samperio P., Miranda E., Trejo A. (2006). Mycobacterium bovis Bacillus Calmette-Guerin (BCG) stimulates human beta-defensin-2 gene transcription in human epithelial cells. Cell Immunol..

[30] Lang P.O., Govind S., Bokum A.T., Kenny N., Matas E., Pitts D., Aspinall R. (2013). Immune senescence and vaccination in the elderly. Curr. Top. Med. Chem..

[31] Goronzy J.J., Weyand C.M. (2013). Understanding immunosenescence to improve responses to vaccines. Nat. Immunol..

[32] Aging NIo (2023). Vaccinations and Older Adults. https://www.nia.nih.gov/health/immunizations-and-vaccines/vaccinations-and-older-adults.

[33] (2011). Ministry of health and family welfare NDDGoHS, MOHFW, Government of India. National Program for Health Care of the Elderly (NPHCE). Operational Guidelines 2011. https://mohfw.gov.in/?q=major-programmes/Non-Communicable-Diseases/Non-Communicable-Diseases-1.

[34] (2011). Central Statistics Office Ministry of Statistics and Programme Implementation GoI. Situation Analysis of the Elderly in India.

[35] Pavan Kumar N., Padmapriyadarsini C., Rajamanickam A., Marinaik S.B., Nancy A., Padmanaban S., Akbar N., Murhekar M., Babu S. (2021). Effect of BCG vaccination on proinflammatory responses in elderly individuals. Sci. Adv..

[36] Kumar N.P., Padmapriyadarsini C., Rajamanickam A., Bhavani P.K., Nancy A., Jeyadeepa B., Selvaraj N., Ashokan D., Renji R.M., Venkataramani V. (2021). BCG vaccination induces enhanced frequencies of dendritic cells and altered plasma levels of type I and type III interferons in elderly individuals. Int. J. Infect. Dis..

[37] Mehta K., Sharma P., Mujawar S., Vyas A. (2022). Role of Antimicrobial Peptides in Treatment and Prevention of Mycobacterium Tuberculosis: A Review. Int. J. Pept. Res. Ther..

[38] Arranz-Trullen J., Lu L., Pulido D., Bhakta S., Boix E. (2017). Host Antimicrobial Peptides: The Promise of New Treatment Strategies against Tuberculosis. Front. Immunol..

[39] Rivas-Santiago B., Schwander S.K., Sarabia C., Diamond G., Klein-Patel M.E., Hernandez-Pando R., Ellner J.J., Sada E. (2005). Human beta-defensin 2 is expressed and associated with Mycobacterium tuberculosis during infection of human alveolar epithelial cells. Infect. Immun..

[40] Jacobo-Delgado Y.M., Rodriguez-Carlos A., Serrano C.J., Rivas-Santiago B. (2023). Mycobacterium tuberculosis cell-wall and antimicrobial peptides: A mission impossible?. Front. Immunol..

[41] Ferrucci L., Fabbri E. (2018). Inflammageing: Chronic inflammation in ageing, cardiovascular disease, and frailty. Nat. Rev. Cardiol..

[42] Caraux-Paz P., Diamantis S., de Wazieres B., Gallien S. (2021). Tuberculosis in the Elderly. J. Clin. Med..

[43] Piergallini T.J., Turner J. (2018). Tuberculosis in the elderly: Why inflammation matters. Exp. Gerontol..

[44] Dourado I., Rios M.H., Pereira S.M., Cunha S.S., Ichihara M.Y., Goes J.C., Rodrigues L.C., Bierrenbach A.L., Barreto M.L. (2003). Rates of adverse reactions to first and second doses of BCG vaccination: Results of a large community trial in Brazilian schoolchildren. Int. J. Tuberc. Lung Dis..

[45] Rodrigues L.C., Pereira S.M., Cunha S.S., Genser B., Ichihara M.Y., de Brito S.C., Hijjar M.A., Dourado I., Cruz A.A., Sant’Anna C. (2005). Effect of BCG revaccination on incidence of tuberculosis in school-aged children in Brazil: The BCG-REVAC cluster-randomised trial. Lancet.

[46] Barreto M.L., Pereira S.M., Pilger D., Cruz A.A., Cunha S.S., Sant’Anna C., Ichihara M.Y., Genser B., Rodrigues L.C. (2011). Evidence of an effect of BCG revaccination on incidence of tuberculosis in school-aged children in Brazil: Second report of the BCG-REVAC cluster-randomised trial. Vaccine.

[47] Kumar N.P., Moideen K., Viswanathan V., Sivakumar S., Menon P.A., Kornfeld H., Babu S. (2017). Heightened circulating levels of antimicrobial peptides in tuberculosis-Diabetes co-morbidity and reversal upon treatment. PLoS ONE.

[48] Tanner R., Villarreal-Ramos B., Vordermeier H.M., McShane H. (2019). The Humoral Immune Response to BCG Vaccination. Front. Immunol..

[49] Kumar N.P., Padmapriyadarsini C., Rajamanickam A., Bhavani P.K., Nancy A., Jeyadeepa B., Renji R.M., Babu S. (2023). BCG vaccination induces enhanced humoral responses in elderly individuals. Tuberculosis.

[50] Kumar N.P., Padmapriyadarsini C., Rajamanickam A., Bhavani P.K., Nancy A., Jayadeepa B., Selvaraj N., Asokan D., Renji R.M., Venkataramani V. (2021). BCG vaccination induces enhanced frequencies of memory T cells and altered plasma levels of common gammac cytokines in elderly individuals. PLoS ONE.

[51] Rajamanickam A., Munisankar S., Dolla C.K., Babu S. (2020). Diminished Systemic and Mycobacterial Antigen Specific Anti-microbial Peptide Responses in Low Body Mass Index-Latent Tuberculosis Co-morbidity. Front. Cell Infect. Microbiol..

[52] de Heredia F.P., Gomez-Martinez S., Marcos A. (2012). Obesity, inflammation and the immune system. Proc. Nutr. Soc..

[53] Pangrazzi L., Naismith E., Miggitsch C., Carmona Arana J.A., Keller M., Grubeck-Loebenstein B., Weinberger B. (2020). The impact of body mass index on adaptive immune cells in the human bone marrow. Immun. Ageing.

